# Progressive Failure Analysis of Thin-Walled Composite Structures Verified Experimentally

**DOI:** 10.3390/ma13051138

**Published:** 2020-03-04

**Authors:** Patryk Rozylo, Miroslaw Ferdynus, Hubert Debski, Sylwester Samborski

**Affiliations:** Faculty of Mechanical Engineering, Lublin University of Technology, 36 Nadbystrzycka Str, 20-618 Lublin, Poland; m.ferdynus@pollub.pl (M.F.); h.debski@pollub.pl (H.D.); s.samborski@pollub.pl (S.S.)

**Keywords:** composite materials, thin-walled structures, carbon-epoxy laminate, progressive failure analysis

## Abstract

The subject of the presented research was a thin-walled composite column made of CFRP (carbon-epoxy laminate). The test sample had a top-hat cross-section with a symmetrical arrangement of laminate layers [90/−45/45/0]_s_. The composite structure was subjected to the process of axial compression. Experimental and numerical tests for the loss of stability and load-carrying capacity of the composite construction were carried out. The numerical buckling analysis was carried out based on the minimum potential energy criterion (based on the solution of an eigenvalue problem). The study of loss of load-carrying capacity was performed on the basis of a progressive failure analysis, solving the problem of non-linear stability based on Newton-Raphson’s incremental iterative method. Numerical results of critical and post-critical state were confronted with experimental research in order to estimate the vulnerable areas of the structure, showing areas prone to damage of the material.

## 1. Introduction

The contemporary engineering approach focuses on the use of modern materials characterized by a high level of load-carrying capacity, with a low weight of the construction. Materials fulfilling this concept belong to the class of materials commonly called composites, which as thin-walled composite columns, are characterized by being prone to the well-known phenomenon of loss of stability [1,2,3,4]. The loss of stability, commonly known as buckling, occurs as a result of the critical load and is a process of transition of the linear operating range of a structure to a non-linear one. Thin-walled composite structures are characterized by the ability to operate after buckling—often up to several times the critical load. Buckling is commonly treated as a defect, but in the vast majority of applications, the possibility of further behavior of the structure in the post-critical state provides a wide range of applications of composite materials in the form of thin-walled structures [5,6,7,8,9,10]. Thin-walled composite structures have found their main application as load-carrying structures in aerospace, construction and automotive industries. The most commonly used types of composite materials are carbon, glass and aramid fiber composites. Due to their high load-carrying capacity, composite structures are most often subjected to axial compression processes. As shown by Samborski [11,12], determination of resistance to damage for laminates can be a challenge, especially in the case of non-symmetric layups. However, a proper mode separation procedures supported with the finite element simulations can precisely foresee and track the interlaminar defect propagation [13].

**Figure 1:**
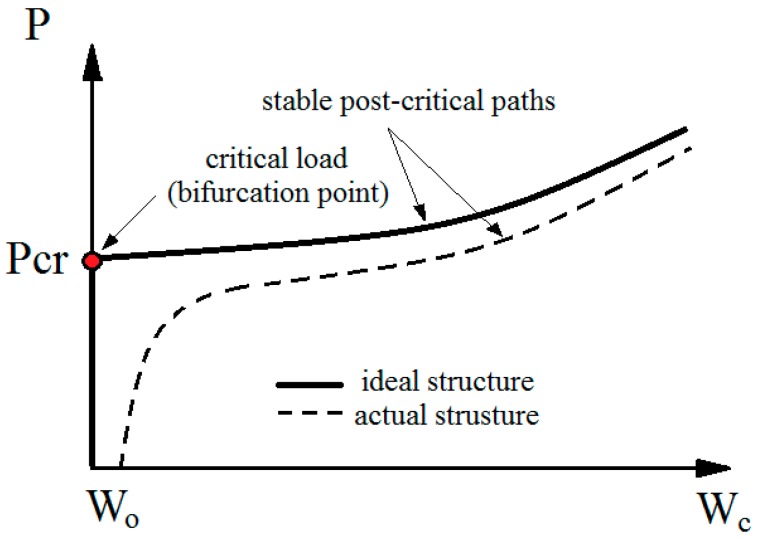
Post-critical equilibrium paths.

Regarding the axial compression of a structure, a typical thin-walled composite structure is characterized by the three main states of work [1,2,9]. The first state, known as pre-critical, is characterized by the structure’s walls only being compressed. The second, critical state, appears after reaching the bifurcation point, as a result of which a linear range of work is transferred to a non-linear one. Post-critical state is the one in which deformations result from bending increases. Post-critical state with great overloading leads to the loss of structural integrity—and thus, to damage. Figure 1 [2] presents a general view of the individual operating states of thin-walled structures, when the post-critical equilibrium path is in stable condition.

**Figure 2:**
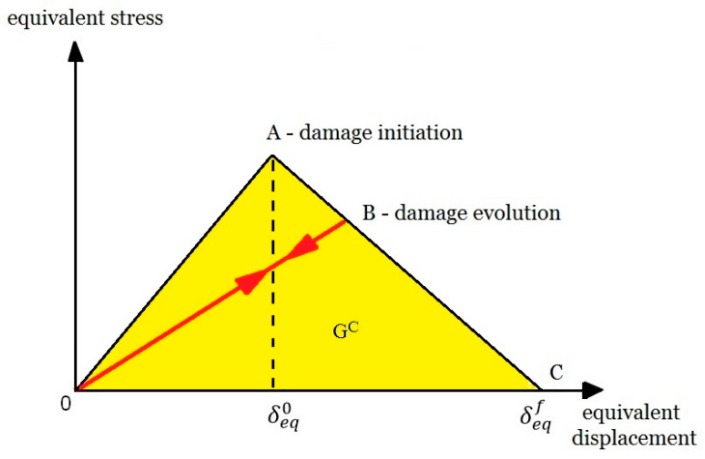
Traction-separation law.

Achieving the bifurcation point is the initial stage of the transition of the structure to work in the post-critical state. Post-critical operation is characterized by certain consequences within the axial compression of the structure. A large number of phenomena associated with permanent damage to the structure, such as fiber cracking, warping or delamination, determine the complexity of the problem of structure failure [14,15,16,17,18]. The studies were conducted over the full load range: at first there is a stage known as the initiation of material damage, followed by the loss of load-carrying capacity—which is directly related to the evolution of the damage. The basic theory for describing the failure of thin-walled composite elements is first ply failure [19]. It is considered that a composite is damaged when the first layer of laminate is damaged. The initiation criterion of Hashin’s failure is widely used, especially in numerical calculations [20]. This criterion makes it possible to assess the state of initiation based on the achievement of initiation parameters of material damage. These parameters are: initiation of damage to stretched and compressed fibers and stretched and compressed matrix. In addition, it is possible to continue the analysis in the following evolution of the damage. In continuum damage mechanics (CDM) the types of damage are represented and modelled by material stiffness degradation [21]—Figure 2.

The paper by Ribeiro [22] presents the development of the damage model and its subsequent application to the simulation of the progressive failure criterion. The work is strictly related to progressive failure analysis (PFA), where the initiation of damage is determined on the basis of Hashin’s criterion discussed earlier [20]. As a rule, for the evolution of damage to take place, it is necessary to meet the initial condition of initiation of damage. The initial approach assumes that damage can be considered as the occurrence of certain micro-cracks by loss of effective cross-sectional area which is caused by micro-cracks—as proposed in his description of damage by Kachanov [23]. This interpretation assumes that the structure’s load is carried only by the undamaged part of the structure’s cross-section. Progressive failure analysis (PFA) requires a declaration of a suitable material model, taking into account the parameters of failure initiation and evolution. The process of stiffness reduction after the initiation of failure takes place according to the model proposed by Matzenmiller [24]. Progressive reduction of material stiffness is controlled by variable failure parameters suitable for failure evolution [25,26,27,28,29,30]. There are five components of the progressive criterion’s parameters responsible for the degradation of material properties: tensile and compressive fiber damage; tensile and compressive matrix; and interlayer shear. All numerical calculations within the cover work of the structure were carried out using Newton-Raphson’s incremental-iteration method [31]. This paper presents an analysis of axially compressed, thin-walled composite structure failure, based on experimental research and numerical calculations performed using the finite element method.

## 2. The Object of Study

**Figure 3:**
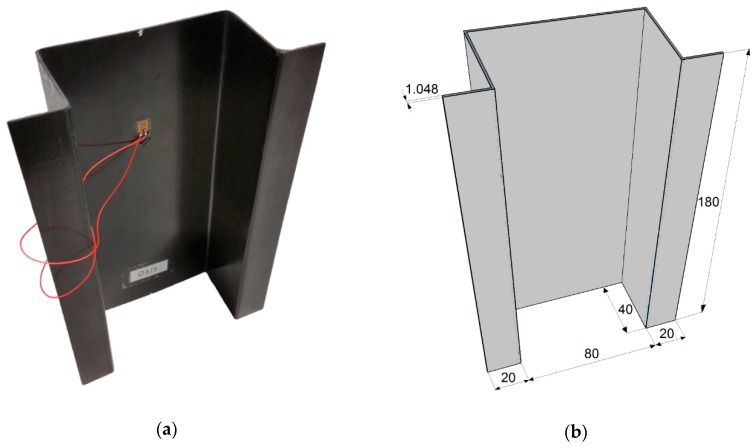
Test specimen: (**a**) actual structure; (**b**) numerical model—sample’s geometry.

The subject of the study was an epoxy carbon composite (CFRP). The test sample was characterized by specific geometrical parameters—Figure 3. The composite structure consisted of eight layers with a symmetrical configuration of their arrangement in relation to the central plane [90/−45/45/0]s. The total thickness of the structure was equal to 1.048 mm.

The material properties determining mechanical and strength parameters of the material are shown in Table 1 [32].

## 3. Research Methodology 

Experimental tests of axial compression were performed on the ZWICK Z100-universal testing machine (ZwickRoell GmbH & Co. KG, Ulm, Germany), under room temperature conditions at a constant velocity of crosshead of 2 mm/min. Special Teflon centering inserts were used to center the sample in the axis of the crosshead displacement. In addition, special plywood panel inserts were used to level the specimen and correct geometric imperfections in the specimen’s end sections. The test sample was fitted with resistance strain gauges located on opposite sides of the web, in the area of the greatest deflections—which was previously estimated on the basis of preliminary numerical analyses. By measuring the strain, it was possible to determine the values of critical load using the Koiter’s approximation method [33,34]. At the same time, measurements of parameters resulting from the acoustic emission (AE) method were performed [35]. The AE equipment was Vallen’s 2-channel AMSY-5 set (Vallen Systeme GmbH, Icking, Germany) with piezoelectric sensors Fujicera 1045S and the AEP-4 preamplifiers. The acoustic emission parameters registered in real time with the maximal sampling frequency of 40 MHz were the numbers of counts and hits, and the amplitude and the energy of the elastic waves released in damage phenomena, along with the deformation of the specimens. An additional facility of the AMSY-5 system was the built-in fast Fourier transform (FFT) tool with upper bound of the frequency range equal 1 MHz, which enabled detection and identification of all the damage phenomena taking place in the CFRP composites: from delamination (tens of kHz) to fiber cracking (hundreds of kHz).

Numerical analysis was performed in ABAQUS^®^ software (Abaqus 2019, Dassault Systemes Simulia Corporation, Velizy Villacoublay, France) based on the FEM. The work used a linear-elastic model, taking into account the orthotropic description of the material model. The discrete model is based on the use of shell elements (S8R) with six degrees of freedom at each of the eight nodes per finite element with reduced integration. The reduced integration technique makes it possible to remove false (erroneous) forms of deformation in finite elements [31]. In the case of non-deformable panels, linear shell elements R3D4 were used. In the process of discrete use, uniform thickening of the finite element grid was ensured by using the structural type of finite elements. The discrete numerical model consisted of 11,620 finite elements and 20,187 computational nodes.

**Figure 4:**
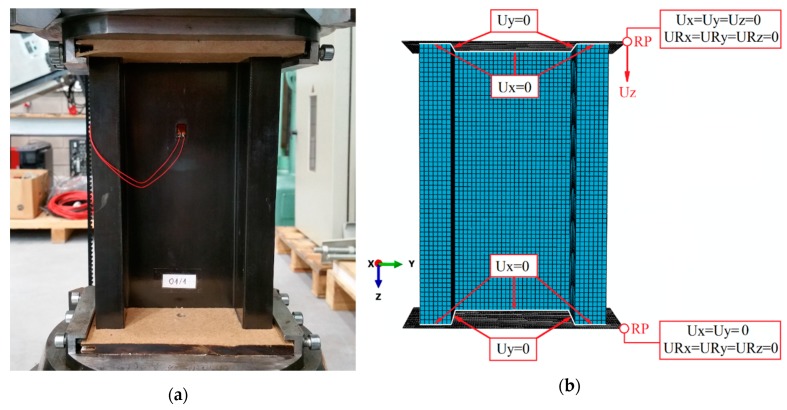
Graphical presentation of boundary conditions: (**a**) experimental study; (**b**) FEM.

Regarding to the discrete model, the boundary conditions corresponding as closely as possible to the experimental research were prepared. In the numerical model, contact properties were applied (in normal and tangential directions, without friction coefficients) between the composite structure and non-deformable plate elements. The plates had defined reference points in which the boundary conditions were determined, which enabled the axial compression tests. Additionally, at the edges of the cross-section of the composite structure, appropriate degrees of freedom were taken away in order for the structure to work properly. Due to the initial implementation of the buckling analysis, a unit force was applied as a load in the numerical model. This approach made it possible to determine the values of critical load as part of the linear solution of the problem (linear perturbation, buckle). Further analysis in the overlapping state was carried out by solving the non-linear structural stability problem, where the unit load was replaced by compressive displacement, performed until the total failure—based on Newton-Raphson’s incremental-iteration method (static, general). Numerical calculations were carried out taking into account preliminary imperfections constituting 0.05 profile wall thickness, which corresponds to the first buckling mode. The boundary conditions of the numerical model and the position for experimental studies are presented in Figure 4.

Numerical and experimental studies were performed for the full range of loads—until total failure. A measurable effect of the experimental tests was to obtain the load-deformation characteristics. The characteristics were obtained on the basis of strain gauge measurements, with the participation of Hottinger measuring set, with the simultaneous support of TestXpert 2 computer system. The introduction of a cover equilibrium path made it possible to assess the load at which the structure loses its load-carrying capacity. The second method enabling the assessment of the damage phenomenon was the measurement of the AE parameters using the piezoelectric sensor based on the AMSY-5 device.

### 3.1. Damage Initiation 

The damage initiation and evolution processes were based on the PFA (primarily on the basis of the Hashin’s criterion, and then on the basis of the energy criterion). Damage of the composite material is usually presented in the literature as a complex, multi-stage process. The composite material is usually closely related to damage of the matrix of the composite material, the cracking or buckling of the fibers and the occurrence of interlayer delamination due to shear stress [35,36]. The level of damage to the composite was determined using progressive damage analysis, which was described by Lapczyk and Hurtado [28]. With reference to PFA, the relationship between the effective stress $\hat{\sigma }$ and nominal stress σ is defined with using the special damage operator M:

(1) $$\hat{\sigma }=M\sigma =\left[\begin{array}{ccc} \frac{1}{1-d_{f}} & 0 & 0\\ 0 & \frac{1}{1-d_{m}} & 0\\ 0 & 0 & \frac{1}{1-d_{s}} \end{array}\right]\sigma$$

where: *d_f_*, *d_m_* and *d_s_* are the specific damage variables for fiber, matrix and shear failure modes, respectively.

The damage initiations of typical, well-known composite materials were defined using the Hashin’s criterion, which has been described in many scientific publications [3,10]. The Hashin’s criterion takes into account four (independent) damage initiation variables: fiber tension or compression and matrix tension or compression. All of damage initiation parameters are described with the following relationships:

(2) $$\mathrm{fiber}\;\mathrm{tension}\;\left(\sigma_{11}\geq 0\right):\;F_{f}^{T}={\left(\frac{{\hat{\sigma }}_{11}}{X^{T}}\right)}^{2}+\alpha {\left(\frac{{\hat{\tau }}_{12}}{2S}\right)}^{2}$$

(3) $$\mathrm{fiber}\;\mathrm{compression}\;\left(\sigma_{11}<0\right):\;F_{f}^{C}={\left(\frac{{\hat{\sigma }}_{11}}{X^{C}}\right)}^{2}$$

(4) $$\mathrm{matrix}\;\mathrm{tension}\;\left(\sigma_{22}\geq 0\right):\;F_{m}^{T}={\left(\frac{{\hat{\sigma }}_{22}}{Y^{T}}\right)}^{2}+{\left(\frac{{\hat{\tau }}_{12}}{2S}\right)}^{2}$$

(5) $$\mathrm{matrix}\;\mathrm{compression}\;\left(\sigma_{22}<0\right):\;F_{m}^{C}={\left(\frac{{\hat{\sigma }}_{22}}{2S}\right)}^{2}+\left[{\left(\frac{Y^{C}}{2S}\right)}^{2}-1\right]\frac{{\hat{\sigma }}_{22}}{Y^{C}}+{\left(\frac{{\hat{\tau }}_{12}}{2S}\right)}^{2}$$

The coefficient α in Equation (2) determines the contribution of the shear stress to the fiber tensile initiation criterion. In the present studies, *α* = 0 was set according to Hashin’s theory (1973) parameter *S^T^* = *Y^C^*/2 (where *S^T^* is transverse shear strength and *Y^C^* is transverse compressive strength). The essence of the presented procedure enables an independent assessment of the damage initiation of fibers and matrix of the composite material.

**Figure 5:**
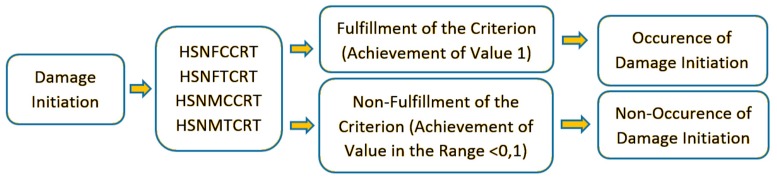
Hashin’s criterion—damage initiation.

The mentioned criterion included four specific components of damage initiation in its description: (fiber tension—HSNFTCRT; fiber compression—HSNFCCRT; matrix tension—HSNMTCRT; and matrix compression—HSNMCCRT). A schematic illustration of the principle of the damage initiation process using the Hashin’s criterion is shown in Figure 5.

In the case of presented procedure, according to numerical calculations, achieving a value of 1 for any component of the criterion is the fulfilment of the damage initiation condition, while obtaining a result in the range <0–1) means that the damage has not been initiated. If the damage initiation criterion is met, the failure may continue (beginning of damage evolution) to evolve as a result of further loading of the structure.

### 3.2. Damage Evolution

Referring to the Hashin’s criterion (damage initiation), the point of damage initiation defines a gradual reduction of stiffness of the material (reduction of mechanical properties of composite material) for a given variable of the damage initiation process. In FEM calculations, damage evolution was described by damage evolution law, which constitutes a generalization of the concept presented by Camanho and Davila [25]. With respect to composite materials, the damage evolution process can be described using exactly five independent damage variables, presented in the theoretical approach: compressive and tensile fiber damage, compressive and tensile matrix damage and shear damage. In the case in which the damage initiation is fulfilled, further loading of the structure causes damage evolution which is realized by fracture energy dissipated during the damage process.

The stiffness matrix of the composite structure can be expressed as the following form:

(6) $$\left\{\begin{array}{c} \sigma_{11}\\ \sigma_{22}\\ \tau_{12} \end{array}\right\}=\frac{1}{D}\left[\begin{array}{ccc} \left(1-d_{f}\right)E_{1} & \left(1-d_{f}\right)\left(1-d_{m}\right)v_{21}E_{1} & 0\\ \left(1-d_{f}\right)\left(1-d_{m}\right)v_{12}E_{2} & \left(1-d_{m}\right)E_{2} & 0\\ 0 & 0 & D\left(1-d_{s}\right)G_{12} \end{array}\right]\left\{\begin{array}{c} \varepsilon_{11}\\ \varepsilon_{22}\\ \gamma_{12} \end{array}\right\}$$

where *σ_ij_* are stresses; *τ_ij_* are shear stresses; *ε_ij_* are strains; *γ_ij_* are shear strains; and parameter *D* is expressed as:

(7) $$D=1-v_{12}v_{21}\left(1-d_{f}\right)\left(1-d_{m}\right)$$

where *d_f_, d_m_* and *d_s_* are the damage variables for fiber, matrix and shear failure modes, respectively.

**Figure 6:**
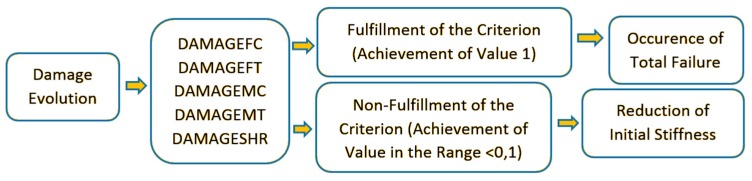
Damage evolution process.

As mentioned earlier, the description of damage evolution for ABAQUS^®^ is initially based on the damage initiation associated with fulfilment the Hashin’s criterion. The main principle of damage evolution assumes that the dissipation energy in the damage process is proportional to the volume of the damaged finite elements of numerical model. In FEM calculations, the damage evolution is strictly controlled by equivalent displacement; therefore, the calculation process itself is based on the constitutive compound “equivalent stress-equivalent displacement” [36]. As mentioned earlier, the progressive damage analysis includes five components, which in the context of numerical analysis are called tensile fiber damage—DAMAGEFT; compressive fiber damage—DAMAGEFC; tensile matrix damage—DAMAGEMT; compressive matrix damage—DAMAGEMC; and shear damage—DAMAGESHR. The algorithm of the progressive criterion is shown in Figure 6.

**Figure 7:**
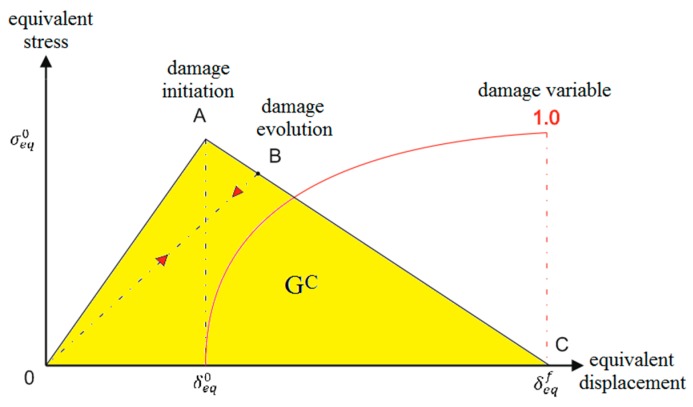
Damage evolution-progressive failure analysis (PFA).

In the case of damage evolution, a value in the range <0–1) means only a percentage loss of initial material stiffness (reduction of material properties). The fulfilment of the progressive failure analysis takes place when the value of 1 for the damage evolution parameter is reached by most of the material failure components. Then it begins loss of load-carrying capacity (until the total failure of the structure made of composite material). An overview of the process of initiation and evolution of damage is shown in Figure 7.

## 4. Results

**Figure 8:**
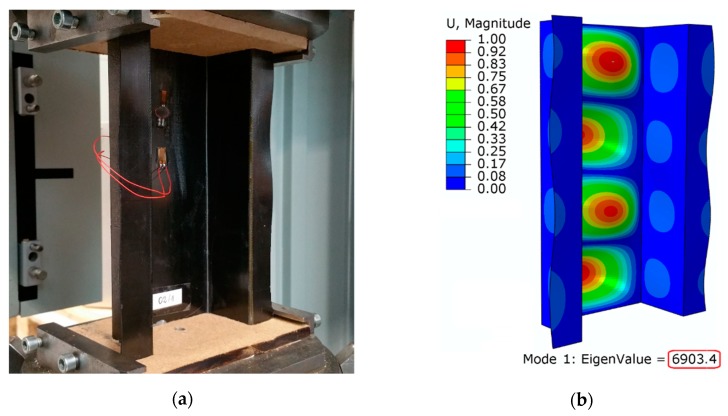
First buckling mode: (**a**) experimental study; (**b**) numerical calculations.

The numerical and experimental research made it possible to evaluate the critical and post-critical condition of the structure. Preliminary analyses were aimed at evaluating the form of deformation of a thin-walled structure at the moment of loss of stability. The numerical calculations estimated that buckling of the composite column occurred with a load of *P_cr_* = 6903.4 N. The buckling mode obtained from experimental and numerical studies is presented in Figure 8.

**Figure 9:**
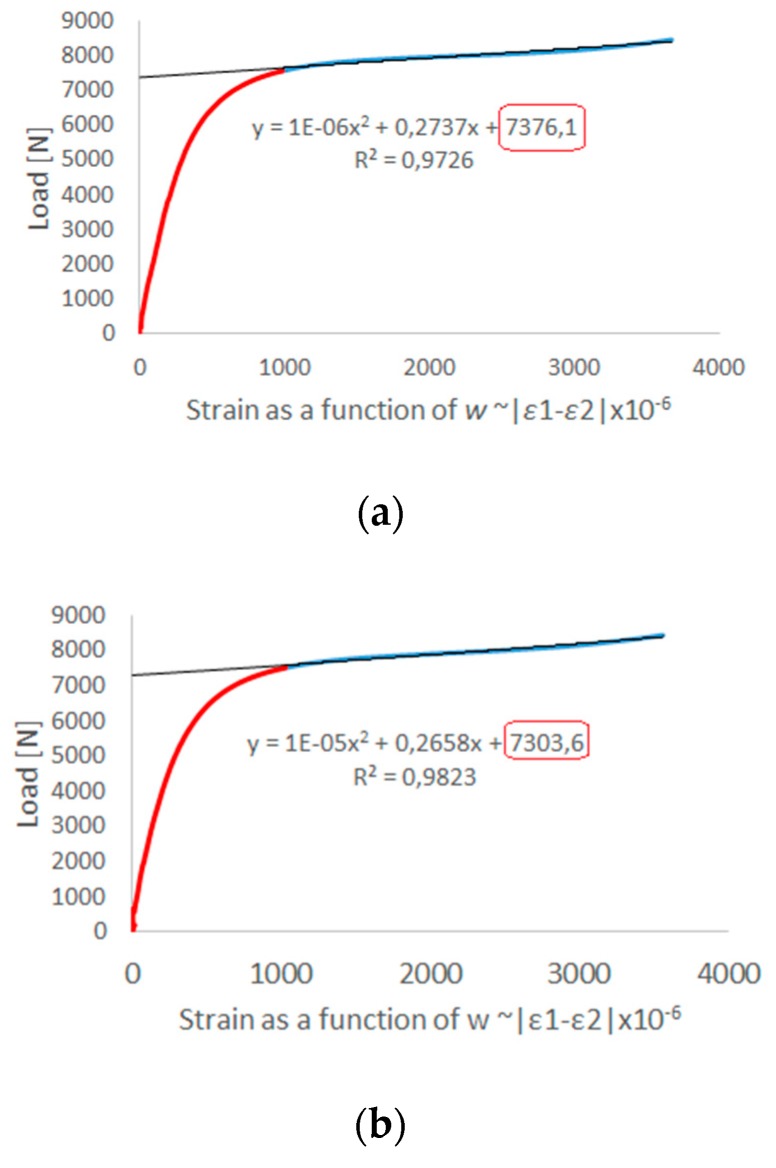

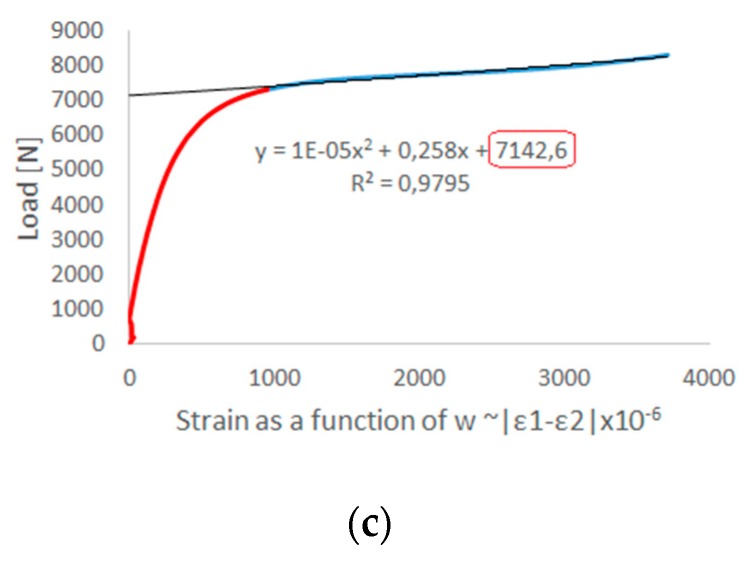
Experimental post-buckling path and approximation line used for determining critical load by Koiter’s method: (**a**) first attempt; (**b**) second attempt; (**c**) third attempt.

In both cases a high correlation was observed, which is the result of the obtained form of deformation of the structure in a critical state, where four half-waves are visible with the loss of stability of the structure. As part of the experimental tests, values of critical load were determined using the second order Koiter’s method. The effective range of the experimental curve was approximated by the second order polynomial. The determinant of the appropriate selection range of the approximated experimental curve was a correlation coefficient in the range of R^2^ ≥ 0.95. Figure 9 shows the determined critical load values as the last components of the approximation functions from three tests.

**Figure 10:**
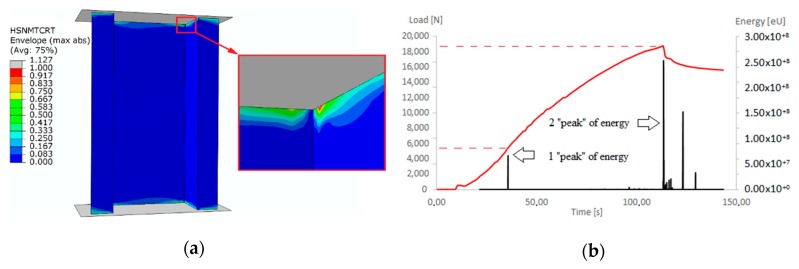
The damage initiation: (**a**) FEM–HSNMTCRT (matrix tension); (**b**) experimental results (first peak of energy—damage initiation, and second peak of energy—damage evolution).

The critical loads determined experimentally maintain the maximum discrepancy at the level of 3.5–6.5%. As part of the analysis of the deep post-critical state, the damage of compressive thin-walled composite columns was identified. Numerical calculations were carried out taking into account the initiation criterion of Hashin’s damage. The applied criterion of failure initiation allowed estimating the load initiating the failure at the level of *P_ini_*_(FEM)_ = 4852 N. The value of the failure initiation load, which was determined experimentally based on the acoustic emission method, was *P_ini_*_(EXP)_ = 5334 N. The discrepancy of presented results was only 9%. Additionally, a map of failure initiation due to tension of the matrix, and the results of experimental study, are presented in Figure 10.

The load initiating the failure process was 30.4% less than the critical load. However, only one of the components of Hashin’s criterion at a low load level reached the initiation (reaching the value of 1 within the initiation parameter). The consequence of the initiation of the failure is the evolution of the failure. At the moment of meeting the conditions resulting from the criterion of damage initiation (fulfilment the initiation for all four components of the criterion), the stiffness of the composite material decreases significantly with further loading of the structure. 

**Figure 11:**
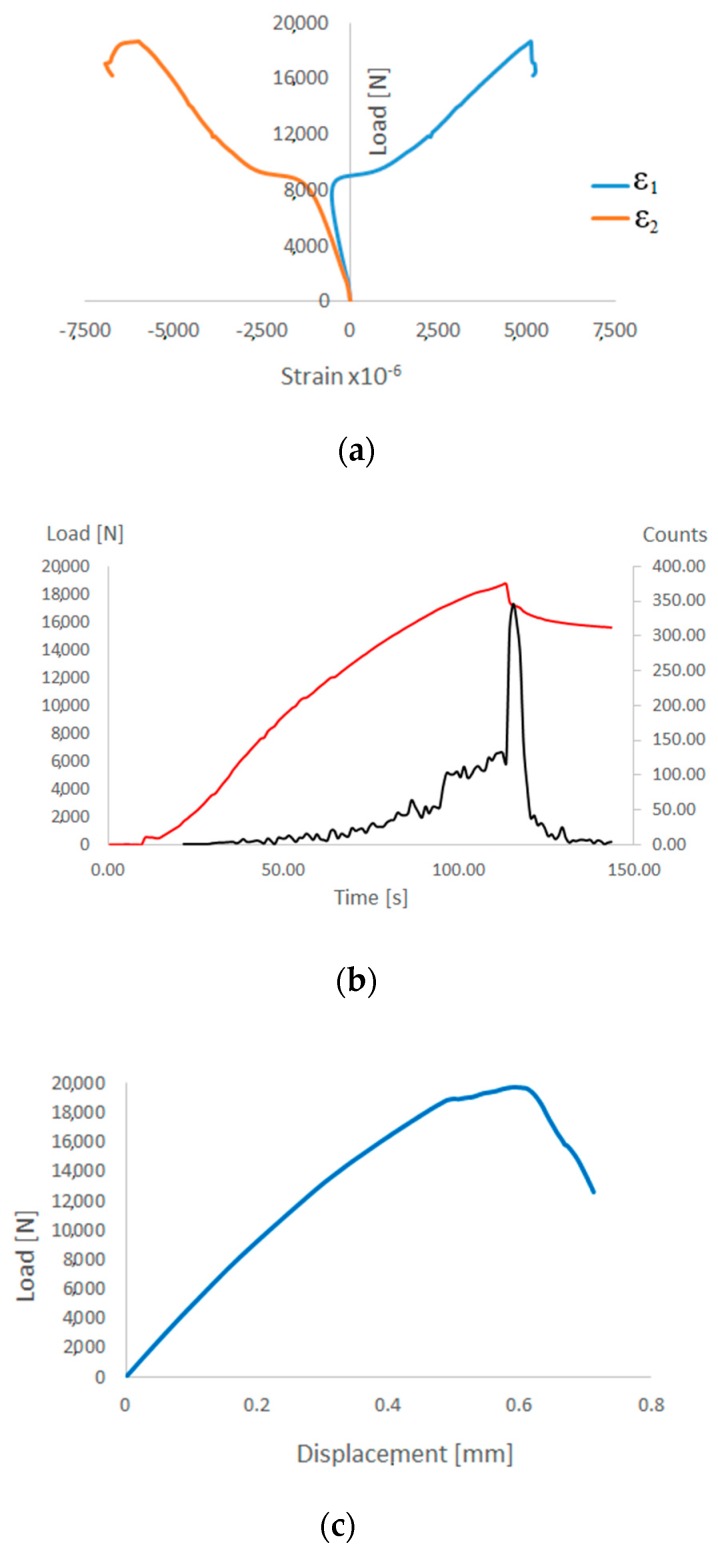
Post-critical equilibrium paths: (**a**) experimental study—strain measurement (strain gauges); (**b**) experimental test—acoustic emission (AMSY-5); (**c**) numerical analysis (FEM).

On the basis of numerical analyses based on the PFA, it was estimated that the value of load causing loss of load-carrying capacity *P_f-FEM_* = 19,723 N. For experimental tests, the load causing a sudden loss of stiffness was *P_f-EXP_* = 18,697 N. The discrepancy between the results of the limit loads was only 5.2%. After achieving one of the four parameters of the Hashin’s criterion, the stiffness of this parameter is gradually degraded according to the scheme shown in Figure 2. Further loads on the structure cause the damage parameter 1 to be achieved by successive parameters of the Hashin’s criterion, which also activates a gradual process of stiffness degradation of these parameters. As a consequence, there is a gradual loss of stiffness of the composite material until the moment of total loss of stiffness of the material. This is a long-term process carried out in numerical calculations using the incremental-iteration method until the moment of losing the ability to carry the load through the compressed structure. In the experimental studies (AEM), a sudden increase in the counts parameter (equal 350) was observed after 120 s of real time, which corresponded to the maximum load (loss of load-carrying capacity) recorded experimentally on a Universal Testing Machine—Figure 11b. An increase in counts may indicate progressive damage (complex state of damage) of the composite structure. The characteristics showing loss of load-carrying capacity in experimental (strain measurement, acoustic emission) and numerical tests are shown in Figure 11.

**Figure 12:**
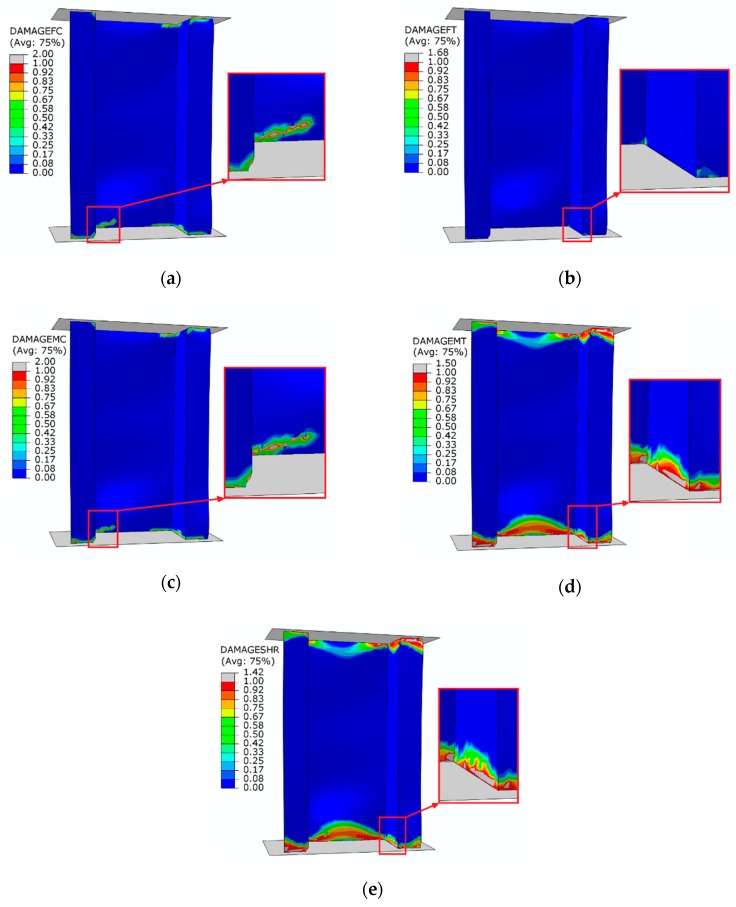
Damage evolution parameters—progressive failure analysis: (**a**) compressed fiber damage; (**b**) tensile fiber damage; (**c**) compressed matrix damage; (**d**) tensile matrix damage; (**e**) shear damage.

Figure 12 presents maps showing the level of damage evolution after total failure of each structure.

**Figure 13:**
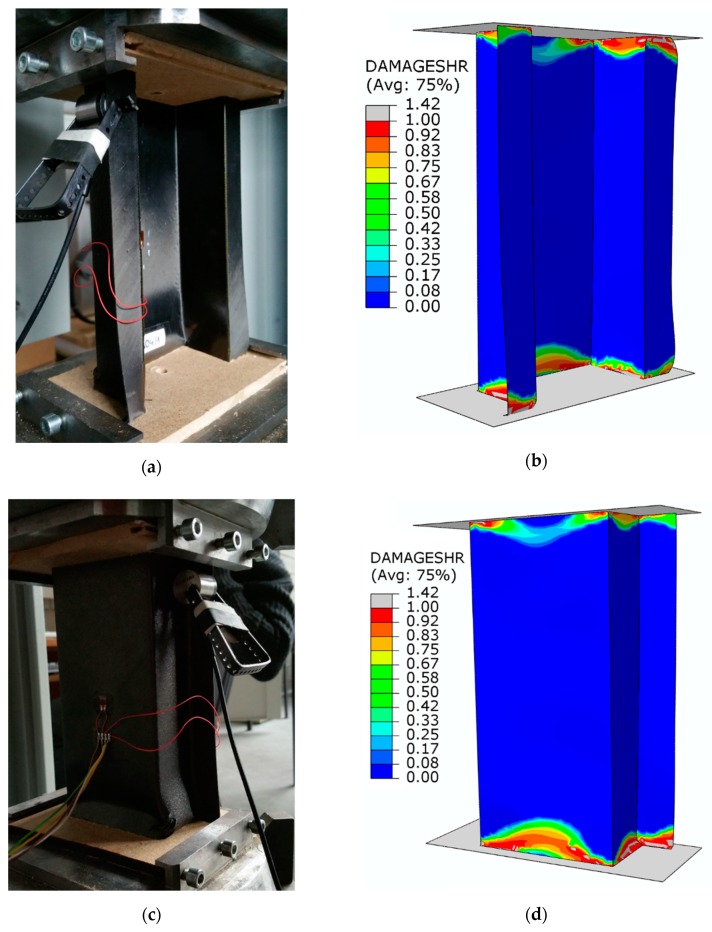
Comparison of the results of failure study: (**a**) experimental test—front side; (**b**) numerical analysis—front side; (**c**) experimental test—back side; (**d**) numerical analysis—back side.

The fulfilment of the damage evolution occurred within all components of the criterion of progressive failure. Red color (reaching the value of 1 within the failure evolution parameter) defines the areas where the failure evolution has been achieved, while grey indicates that the failure evolution has exceeded the nominal value. A graphical comparative presentation of the failure area between experimental tests and FEM is presented in Figure 13.

The damaged area obtained by experimental research corresponded with the results of the numerical simulation. The damage of the material was mainly located in the bottom part of the structure’s cross-section, as a result of which the structure lost its load-carrying capacity. In the tests, a high convergence of the presented results in terms of determining the limit load and the damage area of the material was obtained. As part of the research, high quantitative and qualitative agreement of the presented results was obtained.

## 5. Conclusions

In the study, numerical and experimental tests of a thin-walled composite structure (top-hat cross-section) under axial compression were carried out. Experimental tests were carried out on a universal testing machine-ZWICK Z100. Numerical analysis was carried out with the use of commercial ABAQUS^®^ software. The tests were performed on the full range of loads—until the total failure of the composite structure. As part of the numerical simulation, a progressive failure criterion was used for calculations, which allowed for the degradation of material stiffness due to compression. In numerical studies, non-linear stability analysis of the structure until failure was carried out by using progressive failure analysis-energy criterion (PFA) based on Hashin’s damage initiation criterion. The progressive failure model is a specialized model dedicated in numerical calculations for the analysis of the failure processes of composite materials, allowing for a detailed analysis of the damage of each individual component of the composite material, excluding the debonding process. On the basis of the tests conducted, it was stated that:

- It is possible to determine the values of critical load during buckling of the composite columns;
- Achievement of damage initiation (using Hashin’s criterion) in a given area does not mandate further degradation of material stiffness (progressive failure analysis) in the same structural area;
- It is possible to conduct experimental failure tests based on the measurement of strain gauges and acoustic emission parameters, and numerical damage analysis based on the progressive criterion of material damage.

The obtained convergence of the results of failure load between the experiment and FEM at the level of 5.2% proves the high quality of conducted tests. Macroscopic evaluation of the area of damage located in the bottom part of the cross-section showed the same result for both experimental and numerical studies. 

## Figures and Tables

**Table 1:** Mechanical and strength properties of the composite material.

Mechanical Properties		Strength Properties	
Young’s modulus *E_1_* [MPa]	130,710	Tensile Strength *(0°) F_T1_* [MPa]	1867
Young’s modulus *E_2_* [MPa]	6360	Compressive Strength *(0°) F_C1_* [MPa]	1531
Poisson’s ratio [-]	0.32	Tensile Strength *(90°) F_T2_* [MPa]	26
Kirchhoff modulus *G_12_* [MPa]	4180	Compressive Strength *(90°) F_C2_* [MPa]	214
		Shear Strength *F_12_* [MPa]	100
